# Concussion in the UK: a contemporary narrative review

**DOI:** 10.1136/tsaco-2022-000929

**Published:** 2022-10-19

**Authors:** Emma Toman, Sam Hodgson, Max Riley, Richard Welbury, Valentina Di Pietro, Antonio Belli

**Affiliations:** 1 Institute of Inflammation and Ageing, University of Birmingham, Birmingham, UK; 2 Department of Neurosurgery, University Hospitals Birmingham NHS Foundation Trust, Birmingham, UK; 3 School of Dentistry, University of Central Lancashire, Preston, UK; 4 NIHR Surgical Reconstruction and Microbiology Research Centre, University Hospitals Birmingham NHS Foundation Trust, Birmingham, UK

**Keywords:** review, brain concussion, brain injuries, traumatic, diagnosis

## Abstract

Concussion has been receiving an increasing amount of media exposure following several high-profile professional sports controversies and multimillion-dollar lawsuits. The potential life-changing sequalae of concussion and the rare, but devasting, second impact syndrome have also gained much attention. Despite this, our knowledge of the pathological processes involved is limited and often extrapolated from research into more severe brain injuries.

As there is no objective diagnostic test for concussion. Relying on history and examination only, the diagnosis of concussion has become the rate-limiting step in widening research into the disease. Clinical study protocols therefore frequently exclude the most vulnerable groups of patients such as those with existing cognitive impairment, concurrent intoxication, mental health issues or learning difficulties.

This up-to-date narrative review aims to summarize our current concussion knowledge and provides an insight into promising avenues for future research.

## Definition of concussion

Concussion remains a controversial topic to define within the public sphere and medical community alike. In 2017, the international Concussion in Sport Group (CISG) published a definition of *sports-related* concussion (SRC) as a ‘traumatic brain injury induced by biomechanical forces’, highlighting several common defining features^1^:

1. Injury causes an impulsive force to be transmitted to the head.
2. Rapid onset of short-lived neurological impairment that resolves spontaneously.
3. Neuropathological changes may occur, but the acute clinical symptoms largely reflect a functional disturbance that cannot be seen on standard structural neuroimaging.
4. Graded set of clinical symptoms that may involve loss of consciousness (LOC). Resolution typically follows a sequential course but may be prolonged in some cases.
5. The clinical syndrome cannot be explained by drug, alcohol or medication use, other injuries or comorbidities.

Although these criteria are centred specifically toward SRC, the criteria can be applied to all concussion. Confusingly, the term mild traumatic brain injury (mTBI) is often used synonymously with concussion. Currently concussion is equivalent to ‘uncomplicated’ mTBI where there is no evidence of intracranial injury on CT. ‘Complicated’ mTBI is a subcategory of mTBI where intracranial injury is evident on CT scan and all other mTBI criteria are fulfilled.^2^ First defined by the American Congress of Rehabilitation Medicine (ACRM) in 1993 as ‘traumatically induced physiological disruption of brain function’.^3^ The existing ACRM 1993 criteria for mTBI is compared with several potential revisions suggested by a 2021 expert panel survey in table 1.^4^ As highlighted in table 1, expert opinion supports the use of ‘mTBI’ and ‘concussion’ synonymously as the recommendations for the ACRM mTBI criteria shift the definition closer toward the CISG concussion criteria.

**Table 1:** Comparison of existing ACRM mTBI definition criteria to suggested revisions by 2021 expert panel

1993 ACRM criteria^3^	2021 expert panel suggested revision^4^
A patient with mTBI is a person who has had a traumatically induced physiological disruption of brain function, as manifested by at least one of the following: any period of LOC; any loss of memory for events immediately before or after the accident; any alteration in mental state at the time of the accident (eg, feeling dazed, disoriented or confused); focal neurological deficit(s) that may or may not be transient; but where the severity of the injury does not exceed the following: LOC of approximately <30 min; after 30 min, an initial GCS of 13–15; PTA not greater than 24 hours.	Diagnostic criteria for mTBI should: specify that alternative explanations for altered mental status must be ruled out; incorporate levels of certainty (eg, possible, probable and definite categories) rather than be binary; distinguish between mTBI without; neuroimaging evidence of intracranial trauma; include a maximum timeframe for the onset of symptoms; be expanded to include other observable signs (eg, blank/vacant look or motor incoordination); include a minimum duration of symptoms. Neuroimaging evidence of intracranial trauma implies a more severe form of TBI, not an mTBI. *The terms ‘concussion’ and ‘mild traumatic brain injury’ can be used synonymously.* Rapid-onset postconcussion symptoms after head/neck trauma should indicate at least a possible mTBI.

ACRM, American Congress of Rehabilitation Medicine; GCS, Glasgow Coma Scale; LOC, loss of concisouness; mTBI, mild traumatic brain injury; PTA, post-traumatic amnesia.

## Epidemiology

mTBI accounts for 1.2% of all emergency department (ED) attenders in England and Wales.^5^ The actual number of patients suffering mTBI however are predicted to be much higher as even where concussions are diagnosed or suspected, studies have demonstrated incorrect hospital coding in 90% of cases making health informatics data unreliable.^5^ Furthermore, the Trauma Audit and Research Network, a prospective, observational registry of patients hospitalized in England and Wales following major trauma, is dominated by those suffering mTBI (68%).^6^ This is despite the registry only including patients with 3 or more days in hospital, admission to intensive or high dependency care, interhospital transfer or death from injury, thereby excluding the majority of mTBI.

Young men and older women are at the highest risk of suffering concussion.^7 8^ In the older mTBI population, the predominant sex is female comprising 64% of those aged over 65 years^8^ but overall, it has a male preponderance.^7 9 10^ Assault, falls and road traffic collisions are the most common mechanisms of injury,^6 9^ with falls accounting for the majority of the very young (<15 years) and older (>65 years) populations.^11^

Concurrent comorbidities are common. TBI has long been closely associated with acute alcohol intoxication in those presenting to ED with a head injury.^12^ Similarly, 30% of all patients with mTBI are reported to have psychiatric illness, and circulatory system illness is in >70% of older patients suffering mTBI.^13 14^

Multiple psychosocial factors are also linked to a higher prevalence of concussion including, low cognitive function and social deprivation.^15 16^ Of note, 82% of prisoners have reported sustaining a previous TBI and of these 59% give a history consistent with the diagnosis of concussion.^17^

## Pathophysiology

Knowledge of metabolic, structural and inflammatory processes following brain injury is rapidly evolving and the impact on symptomology and long-term sequelae is an expanding area of research. A summary of changes in the neurometabolic cascade, cerebral blood flow, neuroinflammation and cell structure follows.

### Neurometabolic cascade

#### Ionic flux and neurotransmitter release

Shearing and stretching forces initiate intracellular potassium outflow in central neurons and a subsequent diffuse depolarisation.^18 19^ This is known as mechanoporation and causes a widespread depressive depolarisation leading to immediate postconcussion symptoms.^20^

Compromised gamma-amino-butyric-acid (GABA) interneuron activity allows for exaggerated feedback loops of hyperexcitability and depolarisation due to unregulated glutamate activity.^21^ In animals this is transient and typically normalised within hours.^21^ However, altered glutamate-to-GABA ratios have been seen for days to weeks after concussion in a human sample.^22^

#### Metabolic demand

Increased intracellular sodium and calcium promotes cell damage and mitochondrial impairment,^22^ leading to a metabolic imbalance. This aggravates the cell damage via excitotoxicity, calcium ion overload, reactive oxygen species, Bcl-2 family, caspases and apoptosis inducing factor.^23^ Ineffective metabolism promotes anaerobic energy production and the accumulation of lactate, acidifying the microenvironment.^24^ This is compounded by compromised cerebral blood flow and feeble oxidative breakdown, meanwhile, glucose hypometabolism renders the brain vulnerable to further damage for an unpredictable length of time.^20 24–26^ Better understanding of the influences on metabolic recovery would help to tailor concussion recovery programs.

### Cerebral blood flow and vasculature

#### Cerebral perfusion

Unlike severe TBI, data for dysregulated cerebral autoregulation in mTBI are reliant on advanced imaging, near-infrared spectroscopy or transcranial Doppler, all of which are almost exclusively research tools.^27 28^ Researchers have postulated that the classic triphasic (hypoperfusion, hyperemia and vasospasm) response seen in severe TBI is mirrored in mTBI,^29^ and variable regional, dose and time-dependent changes in cerebral blood flow (CBF) may persist after symptom resolution.^30 31^

#### Blood-brain barrier disruption

The blood-brain barrier (BBB) regulates central nervous system (CNS) connections with the periphery through physical barriers, transport, secretions and metabolic blockade.^32^ In the hours and days following TBI, expression of junctional adhesion proteins decreases leading to greater vessel permeability. The number of endothelial caveolae rise resulting in increased transcytosis of plasma proteins, BBB breakdown and cerebral edema.^33^

In animal models a BBB recovery may be ‘biphasic’, with an early phase disruption, followed by delayed BBB dysfunction days later.^34^ Neuroimaging has similarly shown BBB disruption that takes days to weeks to recover.^35 36^ MRI studies in humans demonstrated a high prevalence of BBB disruption in athletes with repeated ‘subconcussive’ head impacts,^37 38^ and BBB permeability has been identified in 73% of patients with a diagnosis of postconcussion syndrome.^39^ Increased levels of S-100B, a serum biomarker of BBB disruption, show a correlation with poorer neurocognitive and balance scores demonstrating how BBB dysregulation translates to symptomatology.^38^ While there is sufficient evidence to support BBB dysfunction in TBI, there is a paucity of evidence concerning the magnitude and duration of disturbance following mTBI.

### Neuroinflammation

Neuroinflammation in response to concussion has both beneficial and detrimental effects. It occurs via resident glial activation, release of inflammatory cytokines and recruitment of peripheral leukocytes.^40 41^ Microglia produce anti-inflammatory mediators, scavenge cellular debris and choreograph the inflammatory response, yet also produce pro-inflammatory molecules that exacerbate secondary brain injury.^42^ The primary cytokines mediating the inflammatory response following concussion are macrophage inflammatory protein-1α (CCL3), GRO-KC (CXCL1), interleukin (IL)-1α, IL-1β and IL-6.^43^ When the BBB is interrupted, circulating immune cells and plasma proteins capable of inducing neuroinflammation and cerebral vascular dysfunction (including fibrinogen) are more able to extravasate into the brain.^44–46^

A prolonged state of neuroinflammation may linger for years and has been hypothesized to correlate with symptom burden and duration.^47^ Prolonged activation of microglia has been identified up to 17 years following injury and is strongly associated with worsening pathology and outcomes.^48^

### Structural changes

#### Axonal and cytoskeletal injury

Standard neuroimaging cannot detect structural injury following concussion; however, microstructural trauma is thought to play a role in pathophysiology. Diffusion tractography studies have identified multiple areas affected, most frequently the corpus callosum.^49–51^ Many white matter injury measures have been found to correlate with cognitive and neurobehavioral deficits.^51^

Neurofilament and microtubule distortion occurs directly from trauma and secondary to high intra-axonal calcium. This disturbs axonal transport and allows the accumulation of beta-amyloid precursor protein leading to axonal swelling.^52 53^ In vitro stretch injury models demonstrated postinjury axonal undulations, beading and plasma membrane permeability.^53^

#### Neuronal death

Cell death has recently been seen in mild brain injury animal models,^54^ particularly in the hippocampus and thalamus, causing cognitive deficits.^55 56^ Advanced MRI in humans following a single mTBI has demonstrated diffuse volume loss and atrophy of the limbic system and precuneal cortex.^57^ Although this appears to resolve at 1 year, it is correlated with poorer neuropsychiatric testing performance.^58^

Our current understanding of concussion pathophysiology is limited, however, as we explore the interplay of brain metabolism, neuroimmunology, cerebral physiology and structure changing, we will undoubtedly identify more diagnostic and therapeutic targets.

## Diagnosis

No objective test can confirm or disprove a concussive injury. This is a major challenge for diagnosis. In sports, many pitch side tests and metrics aid the gold standard of clinical history and examination, but these are rarely used in non-athlete settings. Advanced imaging, genetic testing and biomarkers are important research tools, but they are yet to prove their utility clinically. There are a vast majority of concussion assessments available^59^; here, the most commonly used concussion tools are discussed.

### Clinical

The clinical picture of mTBI can be broadly subdivided into symptoms, physical signs, balance impairment, behavioral changes, cognitive impairment and sleep/wake disturbance^1^ as shown in table 2.

**Table 2:** Clinical signs and symptoms of concussion

Symptoms	Headache, ‘pressure in the head’, neck pain, nausea or vomiting, dizziness, blurred vision, photophobia, phonophobia, ‘do not feel right’, fatigue, more emotional, irritability, sadness, nervous or anxious, confusion, feeling like ‘in a fog’, feeling ‘slowed down’, difficulty concentrating, difficulty remembering, drowsiness
Physical signs	Loss of consciousness, amnesia, neurological deficit (transient), speech disturbance, lethargy, appears dazed
Balance impairment	Gait unsteadiness
Behavioral changes	Irritability, emotional lability, personality changes
Cognitive impairment	Slowed reaction times, confusion, disorientation
Sleep/Wake disturbance	Somnolence, drowsiness

Diagnosis is straightforward when individuals fall limp to the ground with LOC following head injury, however, this is complicated when the injury is unwitnessed, insidious, without LOC, symptoms are delayed or there is co-existing cognitive deficit or intoxication. Professional rugby has cameras with replay capability and thousands of fans and officials, yet concussions are still missed.^60^ The ED is most frequently the only point of contact with medical services for non-athletes suffering mTBI^7^ and current National Institute for Health and Care Excellence (NICE) guidelines focus on triaging serious head injury to relevant neuroscience or neurosurgery centres,^61^ but lack any advice on how to diagnose mTBI. Moreover, mTBI investigation is often side-lined when there are distracting injuries. For example, over half of patients with a spinal cord injury have a missed TBI and most of these are concussions.^62^

One-third of patients with mTBI in ED have concurrent intoxication,^5^ but the CISG definition of concussion states that ‘clinical signs and symptoms cannot be explained by drug, alcohol or medication use’.^1^ How then are clinicians to diagnose mTBI satisfactorily in these patients? The CISG also states that ‘clinical signs and symptoms cannot be explained by other injuries (such as cervical injuries, peripheral vestibular dysfunction, etc) or other comorbidities (such as psychological factors or coexisting medical conditions)’,^1^ leaving older people and those suffering with cognitive or psychiatric disease more vulnerable to a missed concussion diagnosis.

### Imaging

Both the standard MRI brain protocol (T1, T2, diffusion weighted imaging, susceptibility weighted imaging and fluid-attenuated inversion recovery sequences) and CT head show no abnormality in patients with concussion, as per CISG criteria.

#### Advanced MRI techniques

Diffusion tensor imaging (DTI) is an MRI technique that estimates the white tract anatomy of the brain using anisotropy. DTI shows promise in its diagnostic sensitivity for SRC^63^ through decreases in fractional anisotropy and increase in mean diffusivity and radial diffusivity.^64^ Magnetic resonance apectroscopy has shown promise as neuronal density and viability, glial density, membrane injury and hypoxia are observable through metabolites. Reduced N-acetyl-aspartate and creatine, and a raised choline are most commonly seen.^65^ Such changes have also been identified in clinical concussion studies but appear independent of symptom burden. However, the ability of functional MRI to detect subtle alterations in CBF in response to stimuli or activities in concussed patients does correlate with severity of postconcussion symptomology.^66^ Similarly, arterial spin labeling (ASL) tends to demonstrate decreased CBF in the brains of concussed patients. This technique is, however, relatively new to the mTBI investigation and methodological variations in the current evidence calls for larger-scale ASL studies.^67^

Advanced MRI techniques are able to detect concussion and monitor recovery in research but are almost never used clinically. They are rarely available and when they are, they are highly time consuming, require specialized radiographers and radiologists and are expensive. Overall, these currently outweigh the benefits they present.

### Pitch side tests

The fifth version of the Sports Concussion Assessment Tool (SCAT5) combines symptom, orientation, memory, concentration and balance with clinical neurological examination for assessment of sports mTBI.^68^ Recent investigation into its use in non-athletes showed success in distinguishing concussed from control patients^69 70^ and in Wales, a recent clinical improvement project adapted the SCAT for EDs by removing the sporting reference ‘Maddock’s questions’ and signs at time of injury. The ‘ED-CAT’ project demonstrated that the SCAT system for public healthcare patients in ED is feasible.^71^

### Neuropsychological tests

Neuropsychological (NP) assessment is vital in the management of concussion. This is because the time scale for cognitive recovery may not match that of symptom resolution, therefore, a clinical neuropsychologist is needed.^72^ However, when not available, computerized NP assessments, such as the ‘Immediate Post-Concussion Assessment Tool (ImPACT)’, ‘CogSport’ or ‘Headminer’ may be employed. The ImPACT is the most widely used NP assessment and scores verbal memory, visual memory, reaction time, processing speed and impulse control.^73^ Each composite score is determined through combination of results from a series of cognitive tests (table 3).

**Table 3:** Specific assessments contributing to each domain score of Immediate Post-Concussion Assessment Tool

Verbal memory composite score	Average of these scores: Word Memory total per cent correct (immediate+delay)/2 Symbol Match (hidden symbols)/9×100 Three letters. Total letters correct
Visual memory composite score	Average of the following scores: X’s and 0’s-total correct (interference) total/4 Design memory-total per cent correct (immediate+delay)/2
Reaction time composite score	Average of these scores: X’s and 0’s average correct reaction time (RT) Symbol Match average correct RT/3 Colour Match average correct RT
Processing speed composite score	Average of the following scores: X’s and 0’s-total correct (interference) total/4 Three letters-average counted correctly×3
Impulse control composite score	Sum of the following scores: X’s and 0’s-total incorrect–interference Color match total commissions

The ImPACT scoring requires a pre-injury assessment to which postconcussion scores are compared and a Reliable Change Index is calculated to identify abnormal results.^74^ ImPACT Quick uses percentile scores from large representative samples in place of pre-injury assessment to aid removal-from-play decisions.^75^ Both systems seem promising, however, the need for pre-injury assessment is a clear drawback for use outside of sporting environments. Likewise, ImPACT Quick percentile score calculations exclude individuals with cognitive impairment, learning disability or those over the age of 70 years.^75^ As the majority of those suffering mTBI are older people, this is a clear limitation of the ImPACT in a non-sporting population. In addition, while the ImPACT is available in several non-English languages, not all languages are catered for—an obvious drawback in a multicultural healthcare system.

### Biomarkers

Discovery of a diagnostic biomarker is the holy grail of concussion management. A biomarker can be defined as a naturally occurring characteristic that can be objectively measured and interpreted as an indicator of biological processes or responses to therapeutic interventions.^76^ Ideally, a biomarker of concussion should reflect the level of neuronal injury correlating to brain function and outcome in a linear fashion. The only clinically used biomarker for TBI is S-100β used to determine if a CT scan is warranted for patients following head trauma.^77^ It was incorporated into Scandinavian Neurotrauma Committee TBI guidelines in 2013.^78^ No biomarkers are currently used in the diagnosis or management of concussion.

#### Traditional serum mTBI markers

S-100β is a calcium binding protein found in astrocytes that has been shown to be present in TBI, however, it is not brain-specific and can be found in fatty tissue, bone and skin.^79^ There is some evidence to suggest it correlates with increased incidence of postconcussion syndrome (PCS), MRI changes and NP impairment,^80^ but equally strong evidence to disprove this.^81^ This variability renders S-100β of no clinical use in concussion.

Glial fibrillary acidic protein (GFAP) is a filament protein found within the glial cells of the CNS and have been useful in cerebral infarction, preterm neurological abnormalities, encephalopathy and TBI.^82^ Despite evidence that serum GFAP is elevated in TBI, the focus is on CT-positive TBI or neurosurgical cases and is therefore not relevant to concussion.^81^

Neuron-specific enolase (NSE) is a glycolytic enzyme found in the cytoplasm of neurons, peripheral neuroendocrine tissue and tumors of the amine uptake and degradation system.^83^ Evidence for its use in mTBI is often compromised by poor control groups^81^ and even in severe brain injury, its long half-life makes distinguishing primary from secondary brain injury a difficulty.^84^ Alpha-II-spectrin is the main structural constituent of the cortical membrane cytoskeleton and levels of spectrin breakdown products (SBDPs) have been reported in CSF from adults with severe TBI, demonstrating correlation with clinical outcome.^85^ When compared with injured-control patients, serum SBDPs have been found to be elevated in patients with concussion^86^ and appear to correlate with MR DTI changes and cognitive impairment at 3 months.^87^

Of all the serum biomarkers, ubiquitin carboxy-terminal hydrolase L1 (UCH-L1) shows the most promise. UCH-L1 is a protein found in the neuronal cell body and in a study of 295 patients, was found to be elevated within the first hour of mTBI and appeared to discriminate concussed patients from control cohort.^86^ Importantly, it appears to distinguish patients with brain injury from those with altered Glasgow Coma Scale secondary to drugs and alcohol.^86^

#### Salivary microRNA

MicroRNAs (miRNAs) are small non-coding sections of RNA involved in regulating post-transcription gene expression. Concussion-specific circulating serum miR425-5p was first discovered in 2017 to be downregulated in a small sample of athletes concussed within 1 week compared with athletes concussed within 2 weeks and healthy volunteers.^88^ While serum miRNA shows promise for a diagnostic concussion test, it was felt impractical for the purposes of ‘pitch side’ testing of professional athletes. Saliva has since been suggested as a potential source of miRNA.

Five miRNAs, miR-27b-3p, let-7i-5p, miR-142-3p, miR-107 and miR-135b-5p, have been found to be upregulated in the saliva of concussed players 48–72 hours postinjury compared with matched controls. In addition, this panel of miRNAs showed significant correlation with the reaction time component of the ImPACT assessment.^89^ The most significant results so far came from the Study of Concussion in Rugby Union through MicroRNAs (SCRUM) in 2021 that found a panel of 14 miRNAs that successfully identified concussed rugby players from those with a negative concussion assessment, non-injured controls and musculoskeletal injured controls. The panel was able to differentiate between clinically diagnosed concussion and clinically excluded concussion immediately postmatch and at 36–48 hours. This has significant implications for use in professional sports and for non-athletes in the ED.^90^ Salivary miRNAs are worthy of further investigation in the non-athlete setting where there is a far greater variation in age, physical and cognitive baseline characteristics of patients presenting with head injury.

Tau, neurofilaments, microtubule-associated proteins, cytokines and many more potential biomarkers for mTBI have been researched over the years. In general, study sizes are small, and methodologies are so variable they cannot be compared. There is also wide variation in the results reported. All however agree that a sensitive and specific biomarker for concussion would be a remarkable breakthrough for the management of concussion.

## Sequalae

Concussion recovery usually occurs within a few days. Occasionally, if symptoms persist beyond 10–14 days, PCS is diagnosed. PCS is complex and historically controversial; however, it is now widely recognized in research and clinical practice as a constellation of non-specific post-traumatic symptoms. These may be linked to other coexisting factors separate from ongoing physiological injury. PCS presents variably and symptoms often fall into the somatic, cognitive, psychological/behavioral and sleep subgroups (table 4).

**Table 4:** Common signs/symptoms of PCS

Somatic	Headache, neck pain, dizziness, poor balance, tinnitus, photophobia, phonophobia, diplopia
Cognitive	Poor short-term memory, difficulties concentrating, slowed reaction times, slower mental processing
Psychological/Behavioral	Irritability, change in personality, fatigue, anxiety, depression
Sleep	Insomnia, hypersomnolence, excessive daytime sleepiness

These symptoms have major impact on quality of life. Eighty-two per cent of patients with mTBI in one large prospective study experienced at least one symptom at 6 and 12 months postinjury.^91^ Unfortunately, 40% of the participants demonstrated clinically significant dissatisfaction with life at 12 months using the Satisfaction With Life Score at 12 months postconcussion. Despite media coverage of concussion and PCS in sports, outside of these environments PCS remains problematic to diagnose as symptoms are often non-specific and common to many conditions. Diagnosis also relies on correct original concussion diagnoses which, as previously discussed, is fraught with complication.

## Management

As with all other aspects of concussion, the management of sportspeople and non-athletes is astoundingly different. Groups such as the Rugby Football Union and Football Association implement structured recovery programs focused on rest and graduated return to exercise and contact.^92 93^ The pediatric concussion ethos is to return to education before returning to sport. Yet, in adult non-athlete populations there is almost no structured management of mTBI other than verbal and printed advice that ideally should contain the following^61^:

1. Details of the nature and severity of the injury.
2. Risk factors that mean patients need to return to the ED.
3. A specification that a responsible adult should stay with the patient for the first 24 hours after their injury.
4. Details about the recovery process, including the fact that some patients may appear to make a quick recovery but later experience difficulties or complications.
5. Contact details of community and hospital services in case of delayed complications.
6. Information about return to everyday activities, including school, work, sports and driving.
7. Details of support organizations.

There is only vague, unstructured guidance and the quality of discharge advice is entirely dependent on the hospital delivering it. The UK NICE guidance relies on self-diagnosis of PCS and there is no guidance on mTBI service for delegation of care after discharge. A service like this would be logistically challenging and costly but research suggests early intervention can reduce the likelihood of developing PCS in the huge number of individuals suffering mTBI.^94^ Currently, postconcussion follow-up is inconsistent across national healthcare systems with dedicated concussion clinics in some regions and a stark absence of such provision in others.^95^

The ethos of ‘return to normal activity’ should be applied to all through advice structured around the individuals baseline activity. Advice on sleep, alcohol, driving and time away from work should be implemented alongside targeted medical interventions for early PCS presentation. Programs for returning to premorbid levels of physical activity, or the introduction of physical activity, should be explored from an occupational perspective and as a therapeutic strategy.^96^ Interventions can include pharmacological headache management using non-steroidal anti-inflammatories, beta-blockers, calcium channel blockers, antimigraine medication or peripheral nerve blocks dependent on headache phenotype and associated symptoms.^97^ Adequate NP assessment would help to identify more specific needs for targeted therapies such as treatment of associated affective disorders. In addition, balance and visual assessments may identify the need for focused vestibular, cervical proprioception or oculomotor rehabilitation programs.

## The future

Great advances in knowledge and clinical application have been made in SRC in recent years. While, no doubt, spurred on by litigation initially,^98^ professional sports are taking steps to protect their players and are actively engaging with mTBI research.^90^ Players are beginning to ask questions about their future brain health and awareness of concussion sequalae among the public has never been so pronounced.

As researchers and clinicians there remain many scientific questions unanswered and large gaps in service provision. Given that almost 80% of those presenting to ED with a concussion never have the diagnosis made,^5^ our epidemiological profile of the disease is likely to be incorrect. This leaves studies open to selection bias and the translatability of any findings inapplicable. The majority of investigatory studies in non-athletes actively exclude older people, those with learning disabilities, intoxicated individuals or those with existing neurocognitive issues. As these groups are all at increased risk of head injury, future studies must include such participants if concussion research is to translate into clinical practice.

A specific, objective, diagnostic concussion test would improve detection of mTBI in the ED and give a better refection of which patients are sustaining this injury. This would improve clinical care for those that were previously undiagnosed and would allow the inclusion of traditionally omitted individuals into concussion studies. A non-invasive, point-of-care test is the ultimate goal and salivary miRNA proves a candidate worthy of investigation in non-sports concussion.

Follow-up of patients following a concussion diagnosis is a huge undertaking. The logistical burden of face-to-face follow-up for all individuals renders an outpatient appointment for everyone non-viable. Innovative approaches to follow-up need to be explored. The COVID-19 pandemic forced healthcare to adapt and adopt more remote methods of patient care. Research into such methods should actively involve patients in the design of the service to maximize engagement and improve accessibility.

Future research should also include management strategies for concussed non-athletes. Existing sports recovery programs have the potential to be tailored for public healthcare patients, but it is unlikely that a ‘one-size-fits-all’ approach is suitable for non-sportspeople. Strategies for older people, those with additional educational needs and high burden of comorbidity need to be investigated. In addition, the management of PCS symptoms has to extend to these groups also. Medical therapies that are currently used frequently in young sportspeople may be ineffective outside of this cohort and vestibular rehabilitation strategies may need to be modified for the same reason.

For public healthcare funding to be provided for mTBI services, the true monetary cost of concussion to our society must be calculated. The majority of health-economic studies in mTBI have come from the USA and focus mainly on the acute care expense of mTBI.^99^ As discussed, concussion has far-reaching consequences beyond initial management in the ED. Future health-economic studies must extend beyond emergency care to include the cost of PCS. For example, days lost from work, decreased productivity, additional family doctor/ED attendances, treatments for associated vestibular, somatic, mood disorders and so on. To provide a convincing argument to public healthcare funders, a cost saving must be demonstrated and without true baseline cost data this will not be possible.

Concussion can be a devastating disease and although daunting, further research for non-athletes is crucial if we are to identify and support those suffering in our society.
